# Selective transmission of R5 HIV-1 variants: where is the gatekeeper?

**DOI:** 10.1186/1479-5876-9-S1-S6

**Published:** 2011-01-27

**Authors:** Jean-Charles Grivel, Robin J Shattock, Leonid B Margolis

**Affiliations:** 1 Eunice Kennedy Shriver National Institute of Child Health and Human Development, National Institutes of Health, Bethesda, USA; 2 St George's, University of London, London, UK

## Abstract

To enter target cells HIV-1 uses CD4 and a coreceptor. *In vivo* the coreceptor function is provided either by CCR5 (for R5) or CXCR4 (for X4 HIV-1). Although both R5 and X4 HIV-1 variants are present in body fluids (semen, blood, cervicovaginal and rectal secretions), R5 HIV-1 appears to transmit infection and dominates early stages of HIV disease. Moreover, recent sequence analysis of virus in acute infection shows that, in the majority of cases of transmission, infection is initiated by a single virus. Therefore, the existence of a “gatekeeper” that selects R5 over X4 HIV-1 and that operates among R5 HIV-1 variants has been suggested. In the present review we consider various routes of HIV-transmission and discuss potential gatekeeping mechanisms associated with each of these routes. Although many mechanisms have been identified none of them explains the almost perfect selection of R5 over X4 in HIV-1 transmission. We suggest that instead of one strong gatekeeper there are multiple functional gatekeepers and that their superimposition is sufficient to protect against X4 HIV-1 infection and potentially select among R5 HIV-1 variants. In conclusion, we propose that the principle of multiple barriers is more general and not restricted to protection against X4 HIV-1 but rather can be applied to other phenomena when one factor has a selective advantage over the other(s). In the case of gatekeepers for HIV-1 transmission, the task is to identify them and to decipher their molecular mechanisms. Knowledge of the gatekeepers‘ localization and function may enable us to enhance existing barriers against R5 transmission and to erect the new ones against all HIV-1 variants.

## Introduction

To fuse with the membranes of target cells, human immunodeficiency virus type 1 (HIV-1) uses as receptors two plasma membrane molecules, CD4 and a second one that for historical reasons is called a “coreceptor” [1-3]. For HIV-1 the coreceptor function can be provided by two different receptors, C-C chemokine receptor type 5 (CCR5) or C-X-C chemokine receptor type 4 (CXCR4), these have a normal physiological function serving as chemokine receptors (cytokines). HIV-1 variants that use the CCR5 coreceptor are called R5, those that use CXCR4 are called X4, whilst those that can use both are designated R5X4 (or dual tropic) [4]. Although both R5 and X4 HIV-1 variants are present in body fluids (semen, blood, cervicovaginal and rectal secretions), with a few exceptions R5 HIV-1 appears to transmit infection and dominate the early stages of HIV disease whilst X4 HIV-1 evolves at later stages. If and when this evolution takes place, it is associated with a more rapid loss of CD4 T cells and accelerated progression to the acquired immunodeficiency syndrome (AIDS) (reviewed in [5]).

Until recently it was not clear whether X4 evolves from transmitted R5 as a result of env mutations, or that X4 was initially transmitted but its replication was restricted during the early stages of the HIV disease. Recent genetic studies of HIV-1 variants at the earliest stages of HIV-1 infection have enabled researchers to reconstruct the HIV-1 variants that were initially transmitted [6,7], known as transmitted/founder virus (TF virus). These studies confirmed that only R5, and in a few instances R5X4 HIV-1, but not X4 HIV-1 are transmitted. Therefore, it is reasonable to suggest the existence of a “gatekeeper” that nearly always selects transmission of R5 over X4 HIV-1.

Such a gatekeeping phenomenon may not only select R5 over X4 but may operate among R5 HIV-1 variants as well. Recent studies on TF virus indicate that, in the majority of cases, infection is transmitted by a single R5 viral isolate [6,7]. It was hypothesized that these transmitted R5 virions may have subtle differences that provide advantages for transmission over the majority of R5 virus in biological fluids. If so the gatekeeping mechanism may be even more selective than previously anticipated.

Understanding the scope of potential gatekeeping mechanisms is important not only from the point of view of basic science but also for practical reasons. Indeed, if only selected HIV-1 viruses can transmit infection, microbicides (or preventive vaccines) should specifically target these particular variants, provided that such a strategy will not allow transmission of other HIV-1 isolates. Also, understanding the molecular mechanisms of the selective prevention of transmission of some of the HIV-1 variants, may empower us with the necessary knowledge to expand such gatekeeping to those HIV-1 variants that transmit infection, creating new preventive strategies.

Below, we briefly describe the history of the development of the HIV-1 coreceptor tropism concepts, various patterns of HIV-1 transmission, and the possible mechanisms of gatekeeping.

## R5 and X4 HIV-1: Development of the concept

The controversial history of the discovery of HIV-1 has been the subject of many reviews and has recently been well described by Vahlne [8]. HIV-1 was first called Human T-lymphotropic virus (HTLV-III) or lymphadenopathy-associated *virus* (LAV) by two competing groups. CD4 was shown to be the principle receptor for HIV-1 following a series of observations. These included: the observed decline in peripheral CD4+ T cells in homosexual patients presenting a “newly acquired cellular immunodeficiency syndrome” (AIDS) [9], the ability of HIV-1 isolated from these patients to infect CD4 T cells [10,11], the blockade of HIV-1 infection in vitro by anti-CD4 antibodies [12], and infection of virus-resistant human cells following transfection with the human CD4 gene (hCD4) [13]. However it was found that mouse cell lines transfected with hCD4, were not susceptible to infection. This observation suggested that additional human factors were required for HIV-1 infection. The failure of hCD4 transfected mouse cells to form syncitia (cell-cell fusion) with the HIV-1 producing human H9 cell line suggested that viral entry was the rate limiting step[14]. Furthermore, the inability of hCD4 positive squamous cell carcinoma (SCL1) and astroglial cells (U87MG) to form syncitia with cells expressing HIV-1 envelope demonstrated that infection with the newly-discovered virus required secondary receptor(s) in addition to CD4. These results led to the identification of the HIV-1 co-receptors in 1996. Here, we review some of the work that led to their discovery and the understanding that distinct biological properties of HIV-1 in patients and *in vitro* are due to the existence of distinct viral strains using different cellular coreceptors.

### Envelope polymorphism meets clinical status

The envelope protein gp120 was identified as the viral partner interacting with CD4, and the regions involved in this interaction were mapped by site directed mutagenesis and antibody interference (for review see [15]). Within two years of the discovery of HIV-1, the nature of the interactions between CD4 and the viral envelope gp120 as well as the genetic diversity of HIV-1 isolates, especially in their envelope-encoding gene, were beginning to be understood [16-18]. However, there was no biological correlate for this diversity. The development of diagnostic-tests measuring the presence of the viral core protein p24 [19], anti-HIV-1 antibodies [20-24], and the activity of viral reverse transcriptase (RT) [25], identified HIV-1 infected subjects with a wide spectrum of clinical manifestations. The first attempt to stage the clinical status of HIV-1-infected patients came from Robert Gallo’s group [26] which proposed in 1985 a 6-stage classification of patients positive for gp41 antibodies. This work revealed a linear progression from asymptomatic subjects to AIDS patients. However, the lack of viral isolates at the time prevented any correlation of these different stages of disease progression with *in vitro* biological properties of the virus. It was Eva Maria Fenyo and her associates who first showed that viruses isolated from asymptomatic patients were slow to replicate in *in vitro* culture and yielded low RT levels , while viruses isolated from patients at an advanced stage of disease were fast to replicate and yielded high levels of RT [27]. In this seminal work, the authors noted that “*The relation between the severity of illness and in vitro-replication potential of viruses suggests that during the course of an infection, selection may occur for variants that replicate efficiently in CD4 T cells*”. This “fast/high”, “slow/low” dichotomy of viral isolates constituted the first attempt at classifying the biological properties of HIV-1 and was later confirmed by other groups [28,29] working with patients’ isolates and HIV-1 molecular clones [30-34].

### Extension of the classification of HIV-1 by additional biological properties

Later work identified that many viral isolates from chronically infected patients-induced syncytia, the fusing of HIV-1-infected cells with other CD4 T cells * in vitro *[35] . This was also detected in brain autopsies from HIV-1 infected patients [36]. This gave rise to a second HIV-1 classification based on the ability of certain viral isolates to induce syncytia in culture. Tersmette et al. [37] reported that all viruses isolated from AIDS patients were able to form syncytia in vitro, whereas more than 80% of viruses isolated from asymptomatic patients were not. The former isolates were called syncytia-inducing isolates (SI) and the latter were called non-syncytia inducing (NSI) isolates. SI isolates could be easily differentiated from NSI isolates based on their ability to replicate in H9 cells. The discovery that similar isolates described earlier by Fenyo [27] also showed this phenotype lead to adoption of the SI/NSI dichotomy as an accepted classification for HIV-1 isolates [38-42].

The development of culture methods to differentiate blood monocytes into macrophages susceptible to HIV-1 infection [43] and the use of monocytic indicator cell lines [44] paved the way to the adoption of yet another classification of HIV-1 isolates based on their ability to infect macrophages/monocytes. Isolates able to infect these cells were called macrophage-(M)-tropic. Although M-tropic HIV-1 isolates could not grow in T-cell lines [45], they readily infected primary T cells [46].

As a result, viral isolates were then classified according to their efficiency of replication (fast/high, slow/low), their ability to induce syncytia (SI/NSI) in T cell-lines, and their ability to replicate in T cell-lines or macrophages (T-tropic/ M-tropic). Most primary isolates irrespective of their rapid/high, syncytium-inducing phenotype or slow/low, non-syncytium-inducing phenotype were able to infect both primary T cells and monocyte-derived macrophages *in vitro*, although with unequal efficiencies [47].

### Biological implications of the viral phenotypes

The ability of SI isolates, defined on PBMCs, to replicate in lymphoblastoid T cell lines, H9, MT-2, and MT-4 [48,49], led to the interchangeable use of the terms “SI” and “T- tropic”. Yet, already from the first report describing SI viruses [37] it was clear that the two terms were not interchangeable since not all SI isolates were T-cell-line tropic. Later, the discovery of co-receptors clarified this misconception.

The fact that fast-high, SI, T-tropic viruses were isolated at late stages of disease when patients showed signs of immune suppression and had a declining number of CD4 T cells in their blood, lead to the notion that these isolates were highly cytopathic [37,40,45,50]. In contrast, slow/low, NSI, M-Tropic viruses, isolated initially from asymptomatic patients with no sign of CD4 decline shortly after infection, were thought to be less cytopathic. This notion also turned out to be wrong. In clade B, T-cell-line tropic viruses are only found in 50 % of patients who progress to AIDS [39,49-51] . Furthermore, NSI viruses can be isolated at all stages of HIV disease [39] and therefore caused CD4 T cell decline and disease progression in patients whose viruses did not convert to SI. In addition, the apparent lack of effects of NSI viruses on CD4 T-cell depletion was restricted to peripheral blood [52], while significant CD4 T-cell depletion occurred in the rectal mucosa [53] and duodenum in asymptomatic patients.

### The quest for the co-receptors

Because infection of macrophages by M-tropic virus could be blocked by anti-CD4 antibodies [54], and because the cellular tropism of HIV-1 could be mapped to specific regions of gp120 [55,56], it became clear that this viral protein recognized the same receptor, CD4, on the cell surface of both T cells and macrophages. However, it was proposed that secondary receptors differentially expressed on macrophages and T cell lines might determine infection of these cells by T- and M-tropic virus [57]. The search for these receptors was undertaken using multiple approaches as described above [58-60]. The co-receptor for SI virus was identified by the group of Edward Berger and colleagues [61] as a 7-transmembrane segment G protein-coupled receptor, named “fusin”, This receptor was soon recognized as the CXCR4 chemokine receptor for Stroma cell Derived Factor-1 (SDF-1) [62,63].

The co-receptor for slow/low viruses also turned out to be a chemokine receptor. This became clear in light of a seemingly unrelated publication by Paolo Lusso et al [64] showing that the CC-chemokines Macrophage Inflammatory Protein (MIP)-1α, MIP-1β and Regulated upon Activation Normal T-cell Expressed and presumably Secreted (RANTES) inhibited the replication of M-tropic isolates but not T-tropic isolates. Three different groups [65-67] cloned the CC-chemokine receptor 5 (CCR5), which binds these three CC chemokines and by linking all these studies together, several groups identified CCR5 as being the missing co-receptor for M-tropic viruses [1-3,68]. Thus, in 1996, HIV-1 cellular tropism had finally found a molecular basis: M-tropic viruses (NSI slow/low) were shown to use CCR5 as a coreceptor whereas T-tropic (SI, rapid/high) isolates used CXCR4 [69,70]. Dual tropic viral isolates and their derived clones able to infect primary T cells, T cell lines and macrophages were shown to use both CCR5 and CXCR4 [71]. Although, other chemokine receptors were shown to have HIV-1 co-receptor activity when expressed in cell lines *in vitro* (see Berger et al [72]), it seems that in vivo, only CCR5 and CXCR4 are used to infect cells (with the possible exception of CCR8 [73], and BONZO/STRL33 [74]), The importance of chemokine receptors in HIV-1 infection is demonstrated by (i) an almost absolute [75] resistance to infection of individuals that have a homozygous mutation for CCR5 (delta 32) [76] (although, two cases of infection of such patients by dual tropic CXCR4/CCR5 HIV-1 have been reported [77,78]), (ii) slow disease progression in patient heterozygous for this mutation [79], and (iii) the fact that the switch from CCR5-tropic to CXCR4-tropic HIV-1 is associated with a massive CD4 T cell depletion and a rapid progression to AIDS. However without a switch to X4, R5 HIV-1 variants may undergo mutations that increase their pathogenicity in the course of HIV disease [80].

### A new classification for HIV-1

The discovery of chemokine receptors used by HIV-1 led to the establishment of a new classification of HIV-1 [4] which relies solely on coreceptor usage. As mentioned CCR5-using HIV-1 variants are called R5, whereas CXCR4 using HIV-1 variants are called X4; variants that use both co-receptors are called R5X4. While this classification has been widely adopted, it was also recommended that the type of cells used to passage viral stocks, such as T-cell line-adapted (TCLA) for viruses passaged through a T cell line, should also be indicated. The latter recommendation has somehow been forgotten, which led to a challenge of this monoparametric HIV-1 classification [81]. Moreover, whilst “dual-tropic” HIV-1 variants can utilize both CCR5 and CXCR4 in transfected cell lines, they are often only able to effectively use one of these two coreceptors in tissue displaying either R5 or X4 mono-tropism [82]. Thus, the widely-adopted, monoparametric classification of HIV-1 variants may be modified in the future when more is known about the mechanisms of HIV-1 transmission and pathogenesis.

### A few questions answered

The discovery of HIV-1 coreceptors explained some of the *in vitro* properties of HIV-1 and facilitated rigorous analysis of their role in HIV-1 fusion. This analysis highlighted that all HIV-1 variants have the potential to induce syncitia provided that the target cells express sufficient levels of CD4 and the relevant co-receptor used by that virus. Indeed the reason M tropic viruses did not induce syncytia in H9 and MT2 T cell lines was due to the lack of CCR5 expression on these cells. Furthermore in contrast to the wide distribution of CXCR4 among different subsets of naïve lymphocytes, cells expressing CCR5 constitute a small fraction of peripheral blood T cells limited to memory T cells [83]. In lymphoid tissues CCR5 expression is also confined to a fraction of memory T cells [84] that constitute less than 15% of CD4 T cells [85]. The low abundance of CCR5 expressing CD4 T cells in blood accounts for the apparent low cytopathicity of R5 viruses. However, for cells expressing CCR5, the R5 variants were shown to be as cytopathic as their X4 HIV-1 counterparts for CXCR4-expressing CD4 T cells and were responsible for the depletion of CCR5 expressing CD4 T cells [85]. Accordingly, in the small intestine, where the majority of mucosal T cells express CCR5, infection with CCR5-tropic HIV-1 causes massive depletion of CD4 T cells [53,86-89].

The individual variability in the abundance of CCR5-expressing T lymphocytes [90] and macrophages may determine whether an individual progresses to AIDS with the dominance of R5 HIV-1 or undergoes a switch to X4 HIV-1 that triggers accelerated progression of the disease.

## HIV-1 transmission R5 vs. X4: Gatekeeping

It seems that X4/R5 gatekeeping (that is prevention of X4 HIV-1 from infecting and/or disseminating in the human body at the early stages of HIV-1 infection) belongs to a rare class of almost perfect biological phenomena. Among multiple reported HIV-1 transmission events via sexual acts (between males and females or between males) or through intravenous injection, at the early stages of infection R5 HIV-1 was ubiquitously found. Such precision suggests the existence of a near perfect barrier that selects against X4 HIV-1 transmission. Where are these gatekeepers and what are their mechanisms? There is no definitive answer, however we discuss below potential mechanisms and points at which selection may occur according to the different routes of transmission .

### Intra-vaginal transmission

World-wide the majority of HIV-1 transmission occurs through heterosexual intercourse. Women have increasingly bourn the brunt of HIV infection mainly through vaginal intercourse and this route of infection has been the one most widely studied in various experimental models. In vaginal infection genital mucosa serves as the first port of entry for HIV-1 and the mucosal barrier is probably one of the gatekeepers not only for X4 HIV-1 but for other HIV-1 variants as well [91].

In vaginal intercourse HIV-1 is ejaculated with semen and transverse mucus that covers the mucosa of the lower female genital track. To establish infection HIV-1 needs to access and infect target cells (lymphocytes, macrophages, possibly dendritic cells (DC), and Langerhans cells (LCs) in particular) in the local mucosa, and be transmitted to the draining lymph nodes where it undergoes rapid replication before being disseminated throughout the entire body. It is believed that a major site for HIV-1 transition in the female genital tract is the cervix, especially the endocervix and the transitional zone, which are covered by a single-layered columnar epithelium. Such a layer is less protective against HIV-1 than the stratified epithelia of the vagina [92,93], reviewed in [94]. Also, the endocervix, together with the transition zone, contains a high number of potential cellular targets for HIV-1 [95]. However, HIV-1 genital transmission to women with a congenital absence of a cervix has been reported [96]. SIV has also been transmitted intravaginally to rhesus macaques after hysterectomy [95,97,98].

The fluid of the lower female genital tract which covers the genital epithelia provides the first potential barrier for the virus on its way to dissemination. Female genital fluids are different in different parts of the genital tract: the vagina is covered by an exudate, which enters through the stratified epithelia that cover this organ. The barrier function of this fluid against HIV-1 has not been thoroughly studied. It is believed that vaginal stratified epithelia provides a significant mechanical barrier to many viruses. Furthermore, the exudate is increased with sexual arousal and therefore its composition may significantly change during sexual intercourse. Nevertheless, virus can penetrate the superficial layers of the stratified epithelium and this may be sufficient to reach superficial Langerhans cells and CD4 T cells, and would be enhanced by any micro or macro lesions in this epithelium.

Higher in the genital tract, the epithelia are covered by true mucus produced by cervical secretory cells. Mucin is the main mucus component and in the endocervix it is mainly a product of two genes: MUC4 and MUC5B [99]. Mucus can protect underlying epithelia by two mechanisms: decreasing HIV-1 infectivity via various soluble factors present in it and/or by temporarily trapping virions in the protein mesh, thus slowing their movement by several orders of magnitude compared with water [100-103]. Since HIV-1 is a fragile virus and cannot remain at normal temperature outside of cells for a long time, its infectivity may be significantly decreased if mucus slows viral penetration. Moreover, during vaginal HIV-1 transmission the acidity of mucus is decreased because of the mixture with alkaline semen. This mixture is less viscous than pure mucus, and the diffusion rate of virions in it is only 15 times slower than in water [101]. Nevertheless, this slowing of HIV-1 penetration may be sufficient for significantly reducing HIV-1 infectivity. Since semen contains not only free virions but also lymphocytes that carry HIV-1 [98], trapping these lymphocytes in mucus may also reduce HIV-1 transmission by these carriers.

Chemical defense against HIV-1 is mediated by mucus soluble factors, in particular by chemokines produced by epithelial cells. In some experimental models epithelia was shown to constitutively produce a CXCR4-binding chemokine, SDF-1, thus selectively reducing X4-HIV-1 transmission [104,105]. Also, cervical mucus contains beta defensins that may inactivate HIV-1 on its way to the epithelia (for review see [106]). Defensins are secreted by epithelial cells under the hormonal control of oestradiol and progesterone [107,108]. Some of these defensins are more restrictive against X4 [58,109-112]. However, others do not significantly differentiate between X4 and R5 HIV-1 [109,110]. Microbicidial enzymes, surfactant proteins and complement present in cervical mucus (see[94]), as well as other as-yet unknown soluble factors observed in proteomics experiments [113], may contribute to the gatekeeping effect. As a result, mucus and mucins suppress HIV-1 in inhibitory assays [114]. The two protective mechanisms of mucus may work synergistically, as even temporary trapping of HIV-1 and HIV-1-infected cells provide a longer exposure to the soluble factors present in mucus as well as to anti HIV-1 antibodies that may have been generated in one of the partners.

Additional gatekeeping effects of mucus may be affected by the difference in the surface charges between R5 and X4 HIV-1. The V3 loop in gp120 of X4 HIV-1 has more exposed cationic charge than R5 HIV-1[115].  In principle, this may result in a stronger binding of X4 HIV-1 to the polyanionic mucin and a preferential clearance of these viruses, or at least impairment of their infectivity. However, dilution of cervical mucus by semen may make this effect negligible. The higher cationic charges of X4 HIV-1 gp120 may also make these viruses more prone to bind to heparin sulphate proteoglycans that cover mucosal surfaces and thus may work as a sink for these viruses [116,117]. However, these theoretical considerations regarding the difference between X4 and R5 HIV-1 have to be tested experimentally. Also, it has been shown that agrin, which plays an important role in establishing viral synapses through which HIV-1 can pass from one cell to another, binds preferentially to R5 HIV-1[118].

Another level of complexity in vaginal HIV-1 transmission is that both mucus and cervical tissue characteristics are not constant but rather undergo changes during the menstrual cycle. A window of infectivity at 7 to 10 days post-ovulation, when the defense mechanisms are at a low level, has been identified [119].

Viral particles that go through the cervical mucus reach the epithelial layer. The epithelial layer itself seems to be an efficient mechanical barrier against HIV-1 and other pathogens [95]. Also, epithelial cells of the lower genital tract are not infected by HIV-1 *in vivo*. Although these cells can express coreceptor molecules, they do not in general express the HIV-1 receptor CD4, The possible exception to this rule is the expression of CD4 by uterine epithelial cells at the proliferative phase of the menstrual cycle [120]. However, under laboratory conditions infection of epithelial cells has been reported [121], and a role in HIV-1 transmission has been ascribed to them [122]. Nevertheless, it is believed that for efficient infection it is necessary for HIV-1 to bypass the epithelial layer. This most likely happens through lesions that commonly occur as a result of various infections and probably environmental factors. Also, microlesions may be generated as a result of coitus [123]. Experiments with cervical explants and fluorescence-labeled HIV-1 showed that HIV-1 penetrates genital mucosa similarly to inert particles, that is, via the gaps between cells (T. Hope, personal communication). When virus encounters and infects its first natural cellular targets, predominantly lymphocytes beneath or within the epithelial layer, it may be efficiently disseminated in cell-associated form from cell to cell through viral synapses which seems to be more efficient than cell-free virus transmission [118,124,125]. This early stage of HIV-1 transmission when few founding cells are infected is critical for the further dissemination of HIV-1 through the body [94]. Surprisingly, although R5 HIV-1 readily infects macrophages *in vitro*, the first (founding) infected cells seem to be CD4 lymphocytes [126].

To characterize HIV-1 targets in cervical tissue more thoroughly, it is necessary to apply multi-color flow cytometry. Recently a protocol of cervical tissue dissociation into single cells that retain their antigenic characteristics has been developed [127], thus enabling a thorough analysis of cervical mucosal lymphocytes using flow cytometry [128]. It was found that cervical tissue is particularly rich in CCR5-expressing CD4 lymphocytes, which make this tissue potentially more susceptible to R5 HIV-1 infection than lymphoid tissue [128]. Accordingly, cervico-vaginal explants express a strong gatekeeping function: on average, dramatically more R5 HIV-1 is released from the *ex vivo*-inoculated cervico-vaginal tissues than X4. Moreover, in a relatively small fraction of tissues that were capable of replicating X4 HIV-1 a correlate was found: a higher presence of early differentiated CD4 lymphocytes [128]. The relation of this fraction to the gatekeeping function of cervical tissue remains to be understood.

Earlier, it was reported that CD4+/CCR5+ cells are consistently detected within the stromal papillae that penetrate the epithelial layer; this location probably makes them easy targets for HIV-1 [129]. Also, it was reported that the levels of CCR5 mRNA in the cervix were up to 10-fold higher than those of CXCR4 mRNA [130]. However, opposite results have been also reported [131]. The level of mRNA for surface antigens is not necessarily translated into the level of expressed proteins. It was shown that cervical lymphocytes express not only CCR5 but CXCR4 as well [128].

Beneath and within the epithelial layers are situated DCs and LCs repectively, which may protrude their dendrites through the layer to the lumen. Normally, these cells capture, and process antigens and deliver them to the draining lymph nodes where they present them to T cells. In HIV-1 infection these cells may bind and deliver HIV-1 from the lumen to the draining lymph node. Although it was firmly established that cervico-vaginal LCs are able to transfer HIV-1 in vitro, it is not clear whether these cells can be productively infected *in vivo *[132]. Nevertheless, they may represent one of the significant elements of gatekeeping as these cells express among other HIV-1-binding molecules, CD4 and CCR5 but not CXCR4 [133,134]. Also at various sites these cells may have different levels of expression of various HIV-1 coreceptors, providing gatekeeping for particular viruses. Cells that, upon binding antigens or upon infection with a virus, are capable of moving out of the tissue are collectively called migratory cells [135]. Such cells consist of a heterogeneous population with very different features.

The entire list of such cells is not yet known. Their infection with HIV-1 in cervico-vaginal tissue was demonstrated using explants (see [136]). Macrophages do express CCR5 [137] and are particularly susceptible to R5 HIV-1 infection *in vitro* and *ex vivo*. Thus, they could also play a significant role as gatekeepers against X4 HIV-1, although in tonsillar explants macrophages are infected by X4 viruses [138]. However, studies of early events of infection in non-human primates with SIV did not reveal infected macrophages [126]. Furthermore, TF virus appears to infect macrophages poorly. Whether high expression of CCR5 and early macrophage infection with HIV-1 reflects the situation in humans *in vivo* or represent an “artifact” from using isolates with high macrophage tropism in the explants system, remains to be clarified.

Thus, although cervical mucosa performs gatekeeping functions, it is obvious that this performance is not perfect and that X4 HIV-1 can penetrate this barrier, although much less efficiently than R5 HIV-1.

### Penile transmission

Globally 80% of men acquired HIV-1 infection from vaginal intercourse, i.e. through the penis (see [136,139]). This is also true when infection is acquired through insertive anal intercourse. However, mechanisms of HIV-1 acquisition through the penis are even less understood than for vaginal transmission in women. Nevertheless, it is clear that gatekeeping mechanisms operate for this route of infection, since R5 HIV-1 also starts infection and dominates its early stages in men.

During intercourse the penis comes into close contact with the vagina and is bathed in vaginal fluid. Thus, it is reasonable to assume that all the above-mentioned protective barriers that are associated with female genital tract fluids and that protect cervical epithelia from viral infection, including barriers that are selective for R5 HIV-1, protect penile epithelia as well. As with the vaginal route of infection, genital fluid may be the first “gatekeeping” mechanism in penile infection. In the course of vaginal intercourse, vaginal fluids are mixed with (pre-) ejaculate. The latter increases mucosal pH and also contains various soluble factors that may dramatically change the characteristics of the mucus. Recently, it was reported [124] that such a mixture, unlike each fluid separately, has an inhibitory effect on HIV-1 infectivity. Whether this effect is different for R5 and X4 HIV-1 has not yet been determined.

After penetrating the mucus or the mixture of mucus and semen, HIV-1 reaches the penile epithelium. What part of the penis is the most vulnerable to HIV-1 transmission? Clinical trials performed over the last several years have shown that circumcision greatly reduces the probability of penile HIV-1 transmission. Thus, the foreskin (or other parts of penis that may be indirectly affected by foreskin removal) seems to play a significant role in transmission of HIV-1. The outer foreskin is heavily keratinized and therefore is considered well protected against pathogen penetration. It was shown [124] in *ex vivo* experiments with foreskin explants that it is indeed the case: HIV-1 does not penetrate the outer foreskin well. In contrast, the inner foreskin and frenulum are covered with a much thinner layer of keratin. Accordingly, in *ex vivo* experiments the inner foreskin was shown to be vulnerable to HIV-1 penetration [124]. However, it was recently shown that keratinization of the outer and inner foreskin is not statistically different, and other mechanisms for the differential permeability of these two parts of the foreskin should be considered [140].

During erection the foreskin is stretched out, revealing its inner aspect, which becomes accessible both to cell-free HIV-1 in the vagina as well as to HIV-1-infected cells that may be situated on the surface of the female genital tract. Another factor that may make the foreskin an important portal of HIV-1 entry is the abundance of HIV-1 cellular targets. The entire foreskin is rich in various cells that constitute potential HIV-1 targets including: CD4 T lymphocytes, macrophages, and LCs [133,139,141,142]. In *ex vivo* experiments and in autopsied tissues [139] infection was revealed in CD4 T lymphocytes and in LCs. However, in the outer foreskin these cells are thought to reside beneath the highly keratinized epithelia and are less accessible for HIV-1. In contrast, in the inner foreskin where the keratin layer is thin, LCs are probably the first target cells that HIV-1 encounters. Here LCs are more abundant and nearer the surface and thus cells are more likely to protrude their dendrites through the epithelial layer towards the outer surface.

Foreskin LCs seem to play a significant role in gatekeeping. In experiments with foreskin explants these cells selectively transfer R5 but not X4 HIV-1 to indicator cells. Another gatekeeping process may stem from the fact that the average density of CCR5-expressing cells in the inner foreskin is 10-fold higher than that of CXCR4-expressing cells [139]. However, the extent of individual variations in this parameter remains to be confirmed in a large group of subjects.

Although keratinized epithelium is highly protective, various abrasions, lesions due to STDs, as well as microtrauma, provide access for HIV-1 to the target cells that reside beneath the surface of the organ. Lesions or microtrauma would also render the outer foreskin and/or shaft of the penis vulnerable to HIV-1 infection, providing access to an abundance of cells expressing CD4, as well as CCR5 and CXCR4. However, even when HIV-1 gets direct access to sub-epithelial layers, R5 HIV-1 seems to find more targets than X4 HIV-1 since on average cells expressing CCR5 are situated in the outer foreskin, glans, and frenulum closer to the surface than cells expressing CXCR4. Also the average density of CCR5-expressing cells in the outer foreskin is higher than that of CXCR4-expressing cells [139]. All these features of the foreskin clearly indicate that whether HIV-1 enters through the inner foreskin or through defects in the keratin layer into the outer foreskin, the barriers against infection are selective and protect the foreskin against X4 HIV-1 infection more efficiently than R5 HIV-1.

Although the foreskin seems to be an important site of HIV-1 entry, circumcised men also acquire HIV-1 through the penile route. Thus, other sites of entry besides the foreskin exist. The glans penis in both circumcised and uncircumcised men is covered by highly keratinized squamous epithelia and seems to be relatively protected against HIV-1 entry in the absence of lesions or microtrauma. In contrast, the penile urethra is less protected as it is covered by a non-keratinized columnar epithelium that is narrowly stratified at the meatus and is also populated with CD4 T lymphocytes and macrophages [93]. Both CXCR4 and CCR5 mRNA have been isolated from urethra swabs in equal amounts [143]. However, it is difficult to translate these data into the relative abundance of CCR5- and CXCR4-expressing cells on the basis of the mRNA measurements.

In summary, on its way to dissemination within the body via the penile route of infection, HIV-1 has to overcome many protective barriers. Some of these barriers can discriminate between R5 and X4 HIV-1 and are higher for the latter.

### Gastro-intestinal mucosal transmission

The probability of infection through receptive anal intercourse is much higher than through vaginal or penile intercourse. These data were confirmed in experiments with macaques infected with the same amount of SIV under controlled laboratory conditions [144]. Thus, it seems that fewer protective barriers exist for this route of HIV-1 transmission. Nevertheless, the gastro-intestinal route of transmission also exhibits a gatekeeping mechanism against X4 HIV-1.

The vulnerability of the colorectum to HIV-1 infection stems from two major factors. First, a single layer of columnar epithelium separates the lumen from the inner layers. This layer is fragile and maybe damaged during intercourse. Also, epithelial cells, although not infectible by HIV-1, may be damaged directly by the virus violating the layer’s integrity [145]. Second, lymphocytes in colorectal tissue are constitutively activated, providing HIV-1 cell targets that efficiently replicate virus [90,146,147], facilitating its dissemination. This is probably one of the reasons why this tissue tissue is one of the first that is damaged by HIV-1 infection, irrespective of the transmission route [86,148]. Although fragile and not providing sufficient mechanical protection, colorectal epithelium provides some biological protection. Among other soluble factors it secretes chemokines, in particular stromal-derived factor 1 (SDF-1) [104], the natural ligand for CXCR4 that *in vitro* selectively suppresses X4 HIV-1 but not R5 HIV-1 infection [62].

Even with intact epithelia, HIV-1 may be transmitted through the epithelial layer by transcytosis [105,149] or transferred by DCs [150]. Also, more colorectal CD4 T cells express CCR5 than do tonsillar CD4 T cells [147]. However, colorectal explants can support replication of both R5 and X4 HIV-1, although X4 less efficient, thus the barrier to X4 infection is unlikely to be absolute [90]. Nevertheless, colorectal tissue *per se* seems to be more vulnerable to R5 HIV-1 infection than secondary lymphoid tissue but can be efficiently infected by X4 HIV-1 as well [90].

HIV-1 transmission through oral sex and also mother-to-child transmission are most likely to occur through the gingeva or tonsils. The latter infection is mediated by swallowing HIV-containing fluids in the birth canal or with breast milk. There is a gatekeeping mechanism operating at the upper gastrointestinal site, and again R5 has an advantage over X4 HIV-1 [151-155]. One of the important defense mechanisms in oral sex is the anti-HIV-1 activity of the saliva [156]. Like vaginal mucus, saliva can trap HIV-1 [157]. However, this mechanism does not seem to be selective for R5 and X4 HIV-1. Also, saliva decreases HIV-1 infectivity because of the presence of various soluble factors [158], including proteases [159] and defensins (see [160]). As discussed above, some of the latter suppress X4 more efficiently than R5 HIV-1 providing a basis for another X4 HIV-1 gatekeeping barrier. Vertical transmission mediated by swallowing breast milk may well occur through infection via the tonsils or other lymphoid tissue associated with the Waldeyer’s ring. Indeed exposure of the tonsils to SIV in neonatal macaques is sufficient to establish infection [161].

Another gatekeeping mechanism may be associated with transcytosis and related to the fact that epithelial cells of the small intestine preferentially express CCR5 rather than CXCR4 [147]. Exposure of the small intestine may occur if any virus or infected cells can survive acidification of the stomach, most likely in the first hours to days following birth. It is not clear to what extent this potential pathway for HIV-1 penetration of the epithelial barrier can discriminate between R5 and X4 HIV-1 [105,149]. In *in vitro* experiments it was reported that primary intestinal (jejunal) epithelial cells were able to transfer R5 but not X4 HIV-1 through trancytosis to indicator cells [162].

A few cases of early detection of X4 HIV-1 in vertical transmission have been reported [152,155,163]. However, in these cases it was not clear whether X4 HIV-1 was actually transmitted or evolved from the earlier-transmitted R5 HIV-1. Establishment of a phylogenic relationship between mother’s and child’s viruses is required to distinguish between the two above-mentioned possibilities. In the absence of such work, it was widely believed that the gatekeeping mechanisms of vertical transmission are as tight as those of horizontal transmission. When the phylogenic analysis of the mother-to child transmitted variants has been performed it was found that X4 variants always evolves from the transmitted R5 HIV-1 [164].

However, a recent study has been published that demonstrated the transmission of X4 HIV-1 (and dual-tropic R5X4 variants) from five Ugandian mothers to their babies [165]. As was shown for cases when X4 evolved as a result of R5 HIV-1 mutation, babies with X4 HIV-1 dominance quickly progress to AIDS. In the case referred to above, these babies died earlier than those to whom R5 HIV-1 was transferred [165].

In conclusion multiple mechanisms for preferential transmission of R5 HIV-1 through the gastrointestinal route have been reported. Although the published data are somewhat controversial, these mechanisms may include: preferential secretion of chemokines that bind to CXCR4 rather than CCR5, the higher level of CCR5 expression, and potentially, preferential transcytosis. However, none of these mechanisms alone seems to explain the high efficiency of the gastrointestinal “gatekeeper” in protection of X4 HIV-1 transmission

### Post-mucosal gatekeepers

Following on from the discussion above, although several somewhat efficient gatekeepers exist at the mucosal portals of HIV-1 entry, they are not perfect. Everything we know from studying these gatekeepers indicates that in spite of them, X4 HIV-1 should be capable of entering the human body, although less efficiently than R5 HIV-1. However, *in vivo* X4 infection rarely occurs, except in a few recently reported cases of vertical transmission [165]. Thus, it appears that there are additional post-mucosal gatekeeping mechanisms. This suggestion is supported by the fact that if HIV-1 bypasses mucosal barriers and is delivered directly (intravenously, with a non-sterile syringe needle or with contaminated blood in a blood transfusion) it is again R5 HIV-1 that is transmitted and that dominates early stages of HIV disease.

Since, as described above, R5 HIV-1 was initially thought to be “macrophage-tropic”, macrophages were first considered to be the infected gatekeepers which select for R5 HIV-1 in cases of intravenous HIV-1 transmission when the mucosal barriers are circumvented [166,167]. Although macrophages are thought to be an important HIV-1 reservoir, studies of the early stages of HIV-1 infection indicate that lymphocytes (which do not discriminate between X4 and R5), rather than macrophages, are the first HIV-1 targets [94,95,97,126]. However, macrophages do become infected and these cells along with other antigen-presenting cells (APCs) are less susceptible to cytotoxic T lymphocytes than are infected T cells. This has been clearly demonstrated in animal models [168,169]. As a result of this and other factors, infected macrophages survive longer than lymphocytes, disseminating R5 HIV-1 with which they are predominantly infected. Although the tissue explant model seems to be closer to the situation *in vivo* than isolated cell cultures, it has its own limitations [127]. In laboratories, explant models are infected by HIV-1 suspensions, while *in vivo* it seems that virus is also disseminated from cell to cell through the viral synapses. Through these synapses R5 HIV-1 is selectively transmitted from DCs to resting CD4+ T cells [170].

Finally, it seems that *in vivo* some systemic factors exist that are more restrictive for X4 than for R5 HIV-1. The first candidate for such a factor is the immune response, including the innate one. Both X4 and R5 HIV-1 induce cytokines, including RANTES and SDF-1 [171], that bind to the respective HIV-1 coreceptors and may prevent infection by corresponding X4 or R5 HIV-1. In lymphoid tissue explants X4 triggers secretion of RANTES in concentrations sufficient to suppress R5 HIV-1, however R5 infection does not induce sufficient SDF-1 to suppress X4 HIV-1 infection [171]. Thus, rather than explaining the gatekeeping mechanism, these experiments indicate one of the potential factors responsible for the “switch” of dominance from R5 to X4 HIV-1 at the later stages of the disease.

However, in experiments with rhesus macaques that have been inoculated with both R5 and X4 viruses (SHIV), R5 outcompeted X4 SHIV [169]. Also, reports have shown that one of the conserved gp120-neutralization epitopes [172] is cryptic in R5 but is accessible in X4 HIV-1. These and other immune mechanisms may selectively suppress X4 HIV-1 preventing rapid evolution of X4 from R5 HIV-1. Indeed, R5 and X4 HIV-1 variants have been described that differ from each other by only a few amino acids [173] suggesting they should easily evolve in the absence of any X4 gatekeeping. The *in vivo* mechanisms against X4 HIV-1 are so pervasive that in 50% of individuals infected with clade B HIV-1, X4 never evolves and viruses retain their R5 phenotype despite progression to AIDS [174]. Moreover, although CXCR4-utilizing HIV-1-a variants have been reported for other clades, there is a relative lack of such variants among non-B subtypes (especially of C and D clades) despite aggressive progression of HIV disease [175].

## Transmitted/ founder virus: Another level of gatekeeping?

While the selective transmission of R5 over X4 virus, irrespective of the route of transmission has been recognized for sometime, more recent studies of transmitted founder (TF) virus have suggested that there may be additional levels of gatekeeping amongst R5 viruses themselves. Detailed sequence analysis of virus in acute infection has enabled investigators to determine the sequence of virus associated with transmission [176]. These studies have shown that in <80% of transmissions, infection is initiated by a single TF virus [7,177,178]. This apparent bottleneck can be reduced by inflammation leading to a higher frequency of multi-variant transmissions of 2-10 viruses [91]. Rectal transmission also seems to mitigate the genetic bottleneck observed in cervico-vaginal transmission [179]. However, irrespective of the route of transmission the majority of these isolates are R5, with a few displaying R5X4 phenotype [7,177]. These data reinforce the concept of gatekeeping against X4 virus. Nevertheless, such gatekeepers may not be absolute, as low frequency (4%) X4 transmission has been seen in some studies [165].

More recently there has been an increased focus on determining whether TF virus exhibits certain phenotypic and or molecular signatures. The first striking observation is that while TF viruses are exclusively R5, they show extremely poor infectivity for *in vitro* derived macrophages [7]. While this needs to be confirmed using tissue macrophages these early observations suggest that transmission selects for T cell tropic R5 variants. This phenotypic observation fits with parallel studies in human mucosal tissue explants [90,128,132,180] and studies delineating the initial target cells of infection in the macaque model [94], all of which implicate CD4 T cells as the initial targets for infection. Furthermore, macaque studies suggest that mucosal infection may be dependent upon the ability of TF to infect resting CD4 effector memory T cells [94]. However, whether this represents a gatekeeper against macrophage tropic R5 virus is debatable, as T cell tropic R5 virus is the dominant phenotype in semen of infected individuals and indeed isolation of macrophage tropic HIV-1 in lymph nodes, blood and semen is rare [181].

The second observation, reported in several studies, is the apparent transmission of R5 virus with reduced N-linked glycosylation [182-184]. HIV-1 is known to cloak its envelope protein in N-linked oligomannose to reduce antibody recognition [185]. At first glance such an apparent reduction in glycosylation is perplexing as this potentially renders TF virus more susceptible to antibody neutralization. However such neutralization is not expected to occur for primo HIV-1-exposure and should only hinder HIV-1 transmission in repeatedly exposed recipients. Only a systematic comparison of TF isolated from subjects infected after a single or repeated exposure could answer this question. Therefore there must be additional gatekeepers at play that select reduced glycosylation in spite of the consequential increase in susceptibility to antibody neutralization. Current thinking proposes two possible mechanisms that may not be mutually exclusive. The first relates to the role of N-linked glycosylation in the binding of HIV-1 to C-type lectins. The group of van Kooyk first observed that the C-type lectin DC-SIGN expressed on dendritic cells could bind virus via oligomannose residues expressed on the viral envelope [186]. Such capture of virus can facilitate both direct infection of dendritic cells (cis-infection) and infection of interacting CD4 T cells (trans-infection). This led to the rapid speculation that dendritic cell capture of virus may be an important step in the transmission process. Activation of these cells (potentially by HIV-1 itself) is known to stimulate rapid migration to draining lymph nodes, thereby disseminating virus to an environment rich in activated CD4 T cells [187]. However the interaction of HIV-1 with C-type lectins turned out to be more complex. DC-SIGN is now only one of several C-type lectins (including Langerin) shown to bind HIV-1 and, while under certain conditions this may favor infection, such binding also facilitates viral uptake, degradation and antigen presentation [188]. In vitro only 5-10% of viral uptake by dendritic cells evades destruction by remaining in a tetraspanin-rich compartment but it is unclear whether this accurately reflects processing of virus by tissue dendritic cells. Indeed it has been shown that binding of HIV-1 by the C-type lectin langerin on Langerhans cells provides an efficient mechanism for viral degradation [189]. As these cells are highly abundant in stratified epithelium of genital mucosas, the absence of critical oligomannose residues could provide a selective advantage by avoidance of triggering the cells that define the interface between innate and adaptive immunity. Avoidance of mechanisms that would stimulate cellular and humoral immune responses may be more important to the establishment of infection than the potential increased sensitivity to neutralizing antibodies. Clearly this makes sense in a naïve susceptible population that would have no pre-existing immunity, however this also opens up a window of potential vulnerability to vaccine induced neutralizing antibodies that could be expressed at mucosal surfaces.

An alternative hypothesis has been recently proposed by Anthony Fauci [190]. His team was the first to identify that the HIV-1 envelope of certain viruses could bind to the alpha-4 beta-7 gut homing integrin [191]. While not essential for infection, this integrin is expressed on a subset of highly susceptible CD4 T cells that express high levels of CCR5 and low levels of CXCR4 and are concentrated in intestinal and, to a lesser extent, cervical mucosa. This preferential binding to alpha-4 beta-7 cells is thought to be an important factor in concentrating early viral replication in the intestinal tract of infected individuals, leading to the massive loss of CD4 T cells within this compartment in the first few weeks of infection [192]. Dr. Fauci has proposed that TF virus binds more efficiently to alpha-4 beta-7 cells than those in chronically infected subjects. This difference in binding was shown to be associated with decreased glycosylation within the V1, V2 region of the HIV-1 envelope, putative binding sites for alpha-4 beta-7 [190]. The suggestion being that glycosylation may block the alpha-4 beta-7-mediated virus binding to this highly susceptible population of CCR5 CD4 T cells. Nevertheless, reported differences in N-linked glycosylation patterns between TF virus and later isolates are not universally accepted with a number of studies reporting no reduction in glycosylation [193]. It will be interesting to see whether differences in glycosylation may be influenced by differences in exposure of the native trimer to cellular glycosylation enzymes during *de novo* viral production. Glycosylation may vary according to the phenotype of infected cells [185]. This raises yet another question about transmitted viruses and potential gatekeeping mechanisms as it shifts the spotlight from a virus defined by its genetic make up, to the cells which produced the infecting virus. Despite such apparent discrepancies there is a growing consensus that not all R5 virus are equal in terms of transmission fitness suggesting that there are further gatekeepers to be discovered.

## The multiple barrier principle

Where is the main gatekeeper that selects R5 over X4? As discussed above many multiple barriers have been identified. However, no single barrier appears to explain the almost perfect selection of R5 over X4 in HIV-1 transmission. We suggest that a single “Big Barrier” does not exist [194]. Rather that the superimposition of multiple weak and imperfect barriers is sufficient to protect against X4 HIV-1 infection. Indeed, X4 HIV-1, associated with accelerated progression to AIDS, only appears late in the course of the disease, and then only in 50% of individuals infected with Clade B virus. Indeed the majority of patients progress to AIDS in the absence of X4 evolution. These observations suggest a sustained selective pressure against X4 over R5 replication. A single Big Barrier would be more fragile and breachable by multiple mechanisms including trauma, mutations, transformations etc, likely to provide an all or nothing effect. Thus it seems that sequential barriers of low efficiency would not only be more protective but would provide ongoing suppression of X4 over R5 replication within an infected individual.

**Figure 1:**
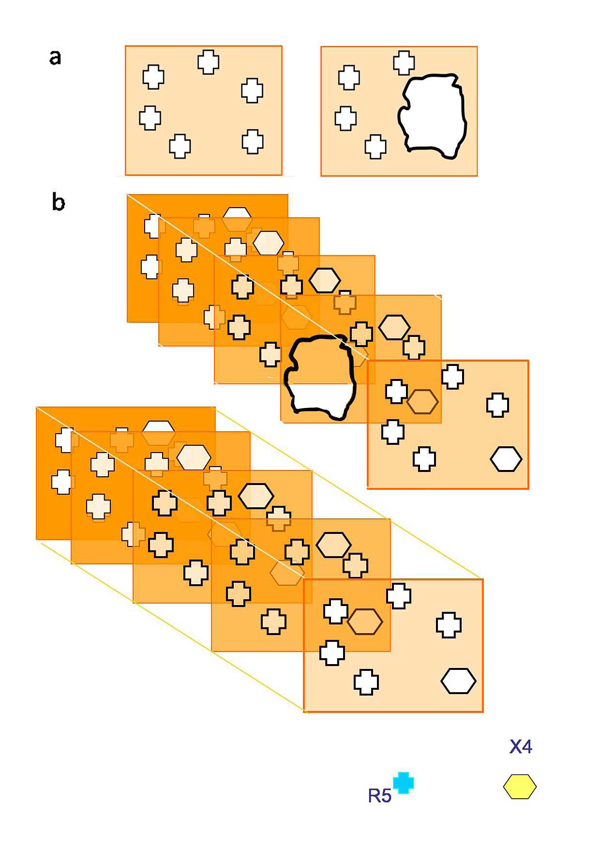
**A set of imperfect multiple barriers provides gatekeeps against HIV-1 better than a single "perfect" barrier (adapted from **[194]**).** (**a**) A "perfect" barrier protects against X4 HIV-1 (left panel). If this barrier is breached (right panel), there is no protection against X4 HIV-1 infection. (**b**) A series of ‘imperfect’ barriers (left panel), each of which protects against X4 virus infection only five times more efficiently than against R5 HIV-1. Nevertheless the chance for X4 HIV-1 to penetrate these barriers is 3125 times lower than for R5. If one of the barriers is breached, (right panel) the system retains relatively high selectivity: The chance for X4 HIV-1 to penetrate the barriers is still 625 times lower than for R5 HIV-1.

This can be illustrated by a simple model consisting of only five sequential barriers each having a selective coefficient of 5 that is the probability ratio for R5 and X4 to penetrate an individual barrier is 5:1. (Figure 1). In this over-simplified construction, although selection of an individual barrier provides only a 5:1 probability of protection against X4 penetration, five sequential barriers provide a probability of 3,125 :1.  Even if one of these five barriers is breached and becomes equally permissive to X4 and R5 HIV-1, the selective power of the construction still remains high at 625:1. It is reasonable to think that the human body has many more than five barriers. Also, their selective power against X4 vs. R5 HIV-1 may be much higher than the 5 described in the above modeling. Although *in vivo* not all of these barriers may be sequential and/or independent, together they are sufficient to protect against X4.

The number of potential barriers may be high and redundant while their efficiency may vary between different individuals. Such a system would make the gatekeeping mechanisms very individualized. Indeed, if in one individual only a few existing barriers against HIV-1 are needed to ensure perfect gatekeeping against X4 HIV-1, in another individual the set of these selective barriers may be different. Nevertheless the net result of the combined gatekeeping of these barriers against X4 HIV-1 would be the same for both individuals. If the number of barriers is large, the huge number of individual variations would make the study of the gatekeeping mechanism very complicated. In this case, identification of individual barriers would require study of large cohorts to reach statistical power.

## Conclusions

In conclusion, we think that the principle of multiple barriers is more general and is not restricted to protection against X4 HIV-1 but rather can be applied to other phenomena when one factor has a selective advantage over the other(s). In the case of X4/R5 gatekeepers, the task of future experiments is to identify each and every one of the selective gatekeepers and decipher their molecular mechanisms. Knowledge of the gatekeepers‘ localization and function may enable us to facilitate existing barriers against R5 transmission and to erect the new ones against all HIV-1 variants.

## Competing interests

The author(s) declare that they have no competing interests.

## Authors’ contributions

J-CG, RS, and LM equally contributed to collecting data, discussing and writing this review.
